# Unveiling the Role of Cholesterol in Subnanomolar Ouabain Rescue of Cortical Neurons from Calcium Overload Caused by Excitotoxic Insults

**DOI:** 10.3390/cells12152011

**Published:** 2023-08-06

**Authors:** Dmitry A. Sibarov, Zoia D. Zhuravleva, Margarita A. Ilina, Sergei I. Boikov, Yulia D. Stepanenko, Tatiana V. Karelina, Sergei M. Antonov

**Affiliations:** Sechenov Institute of Evolutionary Physiology and Biochemistry of the Russian Academy of Sciences, Torez pr. 44, 194223 Saint-Petersburg, Russia; dsibarov@gmail.com (D.A.S.); zhuravlyova@neuro.nnov.ru (Z.D.Z.); magyraccon0909@mail.ru (M.A.I.); sergei-boickov@mail.ru (S.I.B.); juli@unixway.org (Y.D.S.); karelina_tanja@mail.ru (T.V.K.)

**Keywords:** NMDA receptor, neurons, Na/K-ATPase, cholesterol, calcium, Na/Ca exchanger

## Abstract

Na/K-ATPase maintains transmembrane ionic gradients and acts as a signal transducer when bound to endogenous cardiotonic steroids. At subnanomolar concentrations, ouabain induces neuroprotection against calcium overload and apoptosis of neurons during excitotoxic stress. Here, the role of lipid rafts in interactions between Na/K-ATPase, sodium–calcium exchanger (NCX), and N-methy-D-aspartate receptors (NMDARs) was investigated. We analyzed 0.5–1-nanometer ouabain’s effects on calcium responses and miniature post-synaptic current (mEPSCs) frequencies of cortical neurons during the action of NMDA in rat primary culture and brain slices. In both objects, ouabain attenuated NMDA-evoked calcium responses and prevented an increase in mEPSC frequency, while the cholesterol extraction by methyl-β-cyclodextrin prevented the effects. The data support the conclusions that (i) ouabain-induced inhibition of NMDA-elicited calcium response involves both pre- and post-synapse, (ii) the presence of astrocytes in the tripartite synapse is not critical for the ouabain effects, which are found to be similar in cell cultures and brain slices, and (iii) ouabain action requires the integrity of cholesterol-rich membrane microdomains in which the colocalization and functional interaction of NMDAR-transferred calcium influx, calcium extrusion by NCX, and Na/K-ATPase modulation of the exchanger occur. This regulation of the molecules by cardiotonic steroids may influence synaptic transmission, prevent excitotoxic neuronal death, and interfere with the pharmacological actions of neurological medicines.

## 1. Introduction

Sodium–potassium ATPase (NKA) represents one of the most vital enzymes and maintains the transmembrane Na^+^ and K^+^ ionic gradients in all cells that are indispensably required for neuron functioning, considering the ion transport through ion channels, transporters, exchangers, etc. Ouabain is a cardiotonic steroid that specifically binds to NKA and inhibits its pump activity. In many tissues, like cardiomyocytes, renal caveolae, epithelium, and striated and smooth muscles, ouabain inhibits NKA enzymatic activity at concentrations above 50 µM [1]. In contrast to these tissues, neurons, in addition to α1-isoforms, express α3-isoforms of NKA. α1 NKA is many times less sensitive to ouabain than α3 NKA, which is inhibited by ouabain at concentrations above 10 nM [1,2,3]. This property makes neurons more vulnerable to the inhibitory action of ouabain than other cells.

There is a growing body of evidence that suggests NKA also acts as a signal transducer when bound to endogenous cardiotonic steroids at subnanomolar or low nanomolar concentrations [4,5,6,7,8]. Many researchers have reported that cardiotonic steroids have a biphasic effect on NKA ion transport because doses of cardiotonic steroids below IC_50_ enhance NKA activity [9]. For example, at concentrations below 10 nM, ouabain augments NKA ion transport in cardiomyocytes (1 and 10 nM) [2], erythrocytes (0.1 nM) [10], kidney proximal tubule cells [11], and hippocampal slice cultures [12].

It is well established that in many neurodegenerative disorders, an excessive elevation of glutamate may induce neurotoxicity that manifests in neuroinflammation and apoptosis in different brain regions [13]. The prolonged presence of glutamate released from neurons and glial cells via non-quantal secretion [14] and activation of ionotropic glutamate receptors results in intracellular calcium overload and an imbalance in transmembrane ion gradients [15]. As a consequence, activation of multiple pro-apoptotic signaling cascades may occur [16,17]. Similar neuronal dysfunction and neurodegeneration are induced by ouabain concentrations above 0.01 µM, which inhibit NKA enzymatic activity [3,18]. Unlike excitotoxicity, which basically develops through apoptosis, neuroinflammation provides the main contribution to ouabain-induced neurodegeneration [3,18].

Previously, the antiapoptotic action of low ouabain doses was described when kainic acid and ouabain were injected into the rat brain in vivo [19]. At a cellular level, Ca^2+^ overload of neurons caused by agonists of ionotropic glutamate receptors (glutamate, NMDA, L-homocysteine, and kainic acid) is prevented by subnanomolar concentrations of ouabain, which considerably improve neuronal viability [3,20,21,22,23]. As no other binding sites for ouabain are found in animal tissues, except NKA, this effect of ouabain is attributed to signal transduction via NKA [24]. 

Several hypotheses have been proposed recently regarding the mechanism of action of subnanomolar ouabain on intracellular calcium. The downregulation of Ca^2+^ entry through NMDA receptors (NMDARs) due to the local interaction of colocalized NMDAR and NKA is reported [23]. On the other hand, ouabain enhances Ca^2+^ extrusion by the sodium–calcium exchanger (NCX) [20,25], since the inhibition of NCX prevents the ouabain effect in neurons [20]. Moreover, similar to calcitonin gene-related peptide (CGRP) [26], ouabain may cause a delayed effect on neurons by activating multi-kinase neuroprotective signaling cascades [22].

Regardless of the particular mechanism involved in ouabain effects, the colocalization of NKA, NCX, and NMDARs may occur in neurons [27,28,29]. NCX may colocalize and functionally interact with NKA [30,31] and NMDARs [28] in cholesterol-rich membrane lipid raft microdomains. Cholesterol extraction by cyclodextrins may destroy an association between NMDARs and other proteins within the lipid rafts [27,28], which abolishes the interplay between these molecules. If this is the case, the effects realized through NKA, NCX, and NMDAR interactions are predicted to be neglected. For example, this procedure enhances NMDAR calcium-dependent desensitization [25] and weakens the inhibitory effects of tricyclic antidepressants, disrupting the modulation of NMDAR by NCX [32]. 

Therefore, we hypothesized that if the ouabain effects depend on the local interaction between Ca^2+^ entry through activated NMDARs and NCX Ca^2+^ extrusion and its modulation by NKA, the treatment of neurons with methyl-β-cyclodextrin (MβCD) that results in the distraction of lipid rafts may also eliminate the ouabain effects. To achieve this goal, we investigated ouabain action on NMDA-elicited intracellular [Ca^2+^] increase in cortical neurons of primary cultures and brain slices before and after MβCD treatment.

## 2. Materials and Methods

Three main experimental approaches were used to investigate the specific effects of ouabain on intracellular [Ca^2+^] in neurons. Ouabain modulation of somatic Ca^2+^ responses to NMDA was studied using a fluorescent calcium probe. Since the increase in free [Ca^2+^] in pre-synaptic terminals facilitates the generation of miniature excitatory post-synaptic currents (mEPSCs), the frequency of the latter aspect was utilized as an indirect indicator of pre-synaptic [Ca^2+^] variations. The combination of these two approaches allowed us to evaluate the effects of ouabain on both pre- and post-synaptic free [Ca^2+^]. To test the role of glial cells in the ouabain effects, we used the above-mentioned methods to compare the ouabain effects on two experimental objects with a different content of glia, i.e., primary cultures of neurons and brain slices, which were characterized by a deficit and normal composition of glial cells, respectively. The value of lipid microdomains for ouabain effects was assessed through a comparison of the data obtained regarding neurons before and after cholesterol extraction. The experimental protocols are described in more details below.

### 2.1. Animals

All procedures using animals were performed on Wistar rats according to the guidelines of the Federation for Laboratory Animal Science Associations (FELASA) and approved by the Animal Care and Use Committees of the Sechenov Institute. Rats were housed four-to-six per cage at an ambient temperature of 22–25 °C under a 12-h day/night cycle with free access to food and water. For the preparation of primary cultures of cortical neurons, a total of 9 pregnant rats were sacrificed to obtain 26 embryos (approximately two coverslips with culture per embryo were made). To prepare brain slices, 18 infant rats of both sexes were used.

### 2.2. Preparation of Primary Culture of Cortical Neurons

The 17-day pregnant rats were provided by the Sechenov Institute Animal Facility. Animals were sacrificed via 1 min of CO_2_ inhalation in a plastic container connected to a CO_2_ tank. Fetal brains were used to obtain primary cultures of rat cortical neurons using conventional procedures, as described earlier [33]. Neurons were incubated in a “Neurobasal” culture medium supplemented with B-27 (Thermo Fisher Scientific, Waltham, MA, USA) on 15-millimeter glass coverslips coated with poly-D-lysine. Experiments were performed after 10–14 days in culture [33,34].

### 2.3. Preparation of Cortical Brain Slices 

Animals (infant rats at post-natal days 7–9) were decapitated, the brain was rapidly removed, and transferred to ice-cold artificial cerebrospinal fluid (cACSF) of the following composition (in mM): 124 NaCl, 25 NaHCO_3_, 5 KCl, 1.3 CaCl_2_, 1.24 NaH_2_PO_4_, 10 MgCl_2_, 10 D-glucose, 0.5 sodium ascorbate, and 2 sodium pyruvate; the osmolarity was 310 mOsm, and the pH was 7.4. Next, 200-micrometer thick coronal cortical slices were cut using a Leica VT1000S vibration microtome. All ACSF solutions used in experimental procedures were constantly saturated with 95% O_2_ and 5% CO_2_.

### 2.4. Cholesterol Extraction and Repletion

To achieve cholesterol extraction from the plasma membrane of cells, coverslips with neuronal culture or brain slices were treated with 1.5 mM of methyl-β-cyclodextrin (MβCD) for 5 min. In some experiments, coverslips with cultured neurons after this procedure were additionally treated with 0.5 mM of water-soluble cholesterol for 30 min for cholesterol repletion of the plasma membrane.

### 2.5. Calcium Imaging of Cortical Neurons in Primary Cultures and Brain Slices 

Cortical neurons grown on 15-millimeter diameter glass coverslips were loaded with Fluo-8 Ca^2+^-sensitive dye via incubation in the basic solution (in mM: 144 NaCl; 2.8 KCl; 2 CaCl_2_; 10 HEPES, at pH 7.4, osmolarity 310 mOsm) containing 2 µM of Fluo-8 acetoxymethyl ester (Fluo-8 AM) at room temperature for 60 min, followed by a 20-min washout with the basic solution. Then, coverslips were placed onto the stage of the Leica SP5 MP inverted microscope (Leica Microsystems, GmbH, Wetzlar, Germany) and permanently perfused with the basic solution at a flow rate of 1.2 mL/min. Next, 100 µM of NMDA and 0.5 of nM ouabain were applied using a fast perfusion system, which allowed rapid solution exchange. Wherever in the text the concentration of NMDA is indicated, it means that simultaneous application of NMDA and 30 µM of glycine as a co-agonist occurred. Fluorescence was excited using a 488-nanometer laser and detected in a 510–560 nm range with a ~2-second sampling interval (frame 512 × 512 pixels, 20× oil immersion objective). Images were captured using Leica LAS AF v2.3.6 software (Leica Microsystems GmbH, Wetzlar, Germany).

As for brain slices, to enhance Fluo-8 dye penetration into the tissue, the slices were first pre-incubated in cACSF with Cremophor (0.5%) for 4 min at a room temperature of 22–25 °C. Next, the slices were transferred to cACSF containing 2 µM of Fluo-8 AM and Pluronic F-127 (20% solution in DMSO) at 31 °C for 60 min. For astrocyte labeling, the slices were further incubated with 10 µM of sulforhodamine 101 (SR101) for 15 min at room temperature. After these procedures, the slices were stored in sACSF at room temperature for 30 min before experiments occurred. For imaging, the slices were transferred to a Leica TCS SL upright microscope (Leica Microsystems, GmbH, Wetzlar, Germany). The imaging chamber was permanently perfused at a flow rate of 1 mL/min with a 95% O_2_- and 5% CO_2_-saturated sACSF solution. For the application of 100 µM of NMDA and 1 nM of ouabain, a perfusion system allowing rapid solution exchange in the imaging chamber was used. Fluo-8 fluorescence was excited with a 488-nanometer laser and detected in the 510–560-nanometer spectral range with a ~2-second interval for frame capture (frame 515 × 512 pixels, 40× water immersion objective). SR101 fluorescence was excited with a 543-nanometer laser wavelength and detected in a range of 630–700 nm. Images were captured using Leica LCS software (Leica Microsystems GmbH, Wetzlar, Germany).

### 2.6. Patch-Clamp Recording of mEPSCs

Electrophysiological recording of whole-cell currents was performed using a MultiClamp 700B patch-clamp amplifier, which was low-pass filtered at 400 Hz, and digitized at an acquisition rate of 20,000 samples per second using Digidata 1440A and pClamp v10.6 software (Molecular Devices, San Jose, CA, USA). Patch pipettes of 4–6 MΩ were pulled from Sutter BF150-89-10 borosilicate glass capillaries. Experiments were performed at room temperature. Currents were recorded on voltage clamped neurons at −70 mV. For the application of 5 µM of NMDA and 1 nM of ouabain, we used the perfusion system, as described earlier [35]. Mg^2+^ was omitted from experimental solutions to avoid the voltage-sensitive blockage of NMDAR channels [36].

For cortical neurons in primary culture, the external bathing solution contained (in mM) the following components: 144 NaCl; 2.8 KCl; 2 CaCl_2_; and 10 HEPES; these components had a pH of 7.4 and an osmolarity of 310 mOsm. The pipette solution contained (in mM) 120 CsF, 10 CsCl, 10 EGTA, and 10 HEPES; it had an osmolarity in the range of 300–310 mOsm and a pH adjusted to 7.4 with CsOH. 

As for brain slices, the external bathing solution commonly used in experiments (sACSF) contained (in mM) 125 NaCl, 25 NaHCO_3_, 2.5 KCl, 2 CaCl_2_, 1.25 NaH_2_PO_4_, and 10 D-glucose; it was saturated with 95% O_2_ and 5% CO_2_. The pipette solution contained (in mM) 127 Cs-methanesulfonate, 10 NaCl, 10 HEPES, 5 EGTA, 4 ATP-Mg, and GTP-Na; it had a osmolarity in the range of 300–310 mOsm and a pH adjusted to 7.4 with CsOH.

### 2.7. Reagents

These compounds were acquired from Sigma-Aldrich, St. Louis, MO, USA: ATP-Mg cat. A1852; cremophor EL cat. 238470; CsCl cat. C4036; CsF cat. 255718; Cs-methanesulfonate cat. C1426; CsOH cat. 232041; EGTA cat. 324626; glycine cat. G7126; GTP-Na cat. 51120; HEPES cat. 54457; KCl cat. P3911; methyl-β-cyclodextrin cat. C4555; NaCl cat. S9888; NMDA cat. M3262; ouabain cat. O3125; pluronic F-127 cat. P2443; poly-D-lysine cat. P1024; sulforhodamine 101 cat. S7635; and water soluble cholesterol cat. C4951.

Fluo-8 AM Cat. 21080 was obtained from AAT Bioquest, Pleasanton, CA, USA.

Neurobasal medium cat. 21103049 and B-27 supplement cat. A3582801 were acquired from Thermo Fisher Scientific, Waltham, MA, USA.

### 2.8. Data Analysis

For mEPSC detection, we pre-processed the recordings to remove slow baseline fluctuations using the DC-remove function with a time constant of 0.3 s via Spike2 v8 software. This procedure did not affect the amplitudes, shapes, and phases of mEPSCs compared to high-pass frequency filtering. For the detection of mEPSCs, the amplitude of the search threshold in ClampFit software was set as a triple standard deviation calculated for each recording trace.

For each neuron, three samples of mEPSC data were recorded: 10 min as a control, 10 min in the presence of NMDA, and, finally, 10 min in the presence of NMDA and 0.5 nM of ouabain. mEPSC frequency was analyzed in the last 2 min of each of the 10-minute intervals. Recording trace from one brain slice or one coverslip using neuronal culture was considered to be one experiment.

For imaging analysis, the measurements of fluorescence intensity changes over time in regions of interest (ROIs) chosen inside neuronal somas were performed using Leica LAS AF software. The intensity of fluorescence in ROIs in the absence of NMDAR agonists (control) was taken as 1. In brain slices, the cells with intense SR101 staining (red fluorescence) were identified as astrocytes and excluded from the analysis. The average response of neurons from one slice or one coverslip with cell culture was considered to be one experiment.

Quantitative data are expressed as mean ± SEM. GraphPad Prism software v9.0 (GraphPad Software, Inc., Boston, MA, USA) was used for statistical analysis. Student’s two-tailed *t*-test was used to compare groups. The normal distribution of the results was checked via the Kolmogorov–Smirnov test. The number of experiments was indicated by ’n’. The level of statistical significance was set to *p* < 0.05. Data marked by *, **, ***, or **** are significantly different with values of *p* < 0.05, *p* < 0.01, *p* < 0.001, and *p* < 0.0001, respectively.

## 3. Results

### 3.1. Cholesterol Is Obligatory for Ouabain Effects on Neuronal Intracellular Calcium

**Figure 1:**
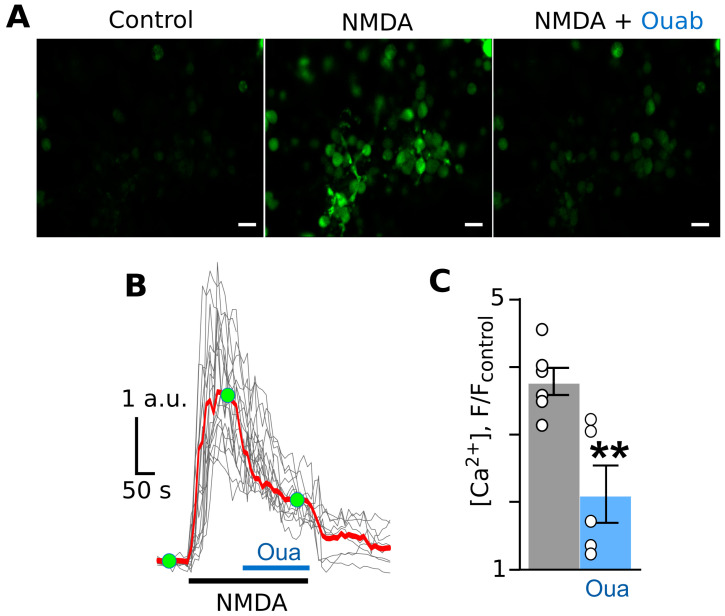
Ouabain causes the attenuation of NMDA-elicited calcium responses in cortical neurons in primary culture. (**A**) Fluorescent images of neurons in the absence of agonists (control), in the presence of NMDA (100 µM of NMDA and 30 µM of glycine), and in the presence of NMDA and 0.5 nM of ouabain. The bar is 10 µm. (**B**) Fluorescent Ca^2+^ responses of neurons evoked by NMDA (100 µM of NMDA and 30 µM of glycine) obtained via a single experiment and normalized to the fluorescence intensity recorded without NMDA (F/F_control_). Gray lines depict responses of neurons in the optic field, where each line is a response of a single neuron. The red line is an average response of neurons. Moreover, 0.5 nM of ouabain was added on top of the NMDA-evoked response. Green circles indicate the time points of intensity measurements picked for statistics. The bottom bars indicate the protocols of the experiments. (**C**) In the histogram, the average amplitudes of Ca^2+^ responses to NMDA before (grey) and after (blue) ouabain application are compared. Data from each experiment (symbols) and mean values ± S.E.M. are shown. **—the data are significantly different (*p* = 0.004, n = 5, Student’s two-tailed *t*-test).

In cortical neurons of primary cultures, an application of 100 µM of NMDA elicited rapid calcium-triggered fluorescent responses, reaching maximal values in 2 min (Figure 1A,B). The addition of 0.5 nM of ouabain on top of the NMDA-evoked calcium response caused a decline in the fluorescence, meaning that at the point of measurement, the Ca^2+^ response amplitude was smaller than that obtained at the beginning (Figure 1C).

**Figure 2:**
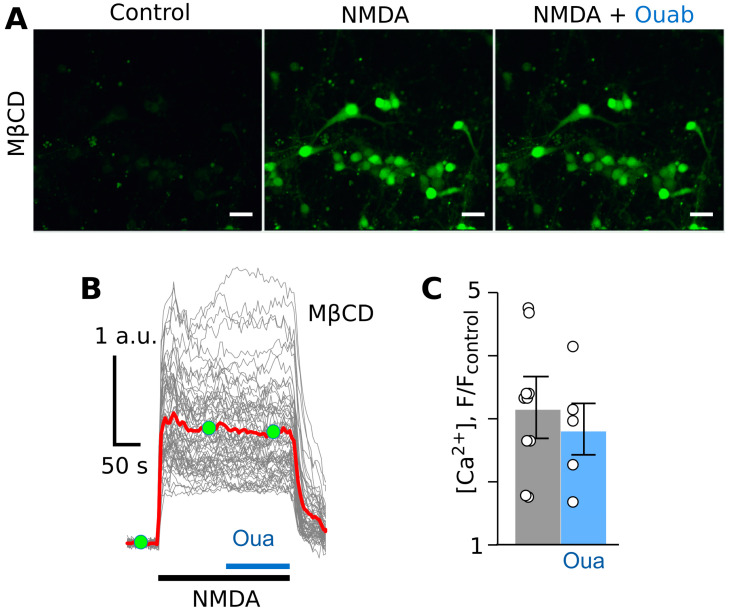
Ouabain do not affect NMDA-elicited calcium responses in MβCD-treated cortical neurons in primary culture. (**A**) Fluorescent images of neurons in the absence of agonists (control), in the presence of NMDA (100 µM of NMDA and 30 µM of glycine), and in the presence of NMDA and 0.5 nM of ouabain. The bar is 10 µm. (**B**) Fluorescent Ca^2+^ responses of neurons evoked by NMDA (100 µM of NMDA and 30 µM of glycine) obtained via a single experiment and normalized to the fluorescence intensity recorded without NMDA (F/F_control_). Gray lines depict responses of neurons in the optic field, where each line is a response of a single neuron. The red line is the average response of neurons. Moreover, 0.5 nM of ouabain was added on top of the NMDA-evoked response. Green circles indicate the time points of intensity measurements picked for statistics. The bottom bars indicate the protocol of the experiments. (**C**) In the histogram, average amplitudes of Ca^2+^ responses to NMDA before (grey) and after (blue) ouabain application are compared. Data from each experiment (symbols) and mean values ± S.E.M. are shown. The data do not significantly differ (*p* = 0.99, n = 5, Student’s two-tailed *t*-test).

To investigate whether the observed ouabain effect depends on the NKA’s association with lipid rafts [37], treatments of neurons with MβCD, which are thought to extract membrane cholesterol and cause disaggregation of lipid rafts in the plasma membrane, were used in experiments [29]. This procedure presumably abolishes the local functional interaction between the lipid raft-associated NMDARs and NCXs [25]. With this aim, experiments were performed on neurons of primary cultures incubated in the basic solution containing 1.5 mM of MβCD for 5 min. While neurons responded to NMDA after this treatment (Figure 2A,B), the addition of ouabain did not cause any noticeable decrease in the NMDA-induced Ca^2+^ responses (Figure 2C), meaning the amplitudes of the responses remained stable. Due to this particular feature, the time course of responses resembled the shape of the NMDA-induced intracellular Ca^2+^ increase recorded in the absence of ouabain on neurons not treated with MβCD [20,21]. It appears that the treatment with MβCD prevents the ouabain effect, which makes the shape of NMDA-evoked intracellular Ca^2+^ responses similar to those obtained without ouabain.

**Figure 3:**
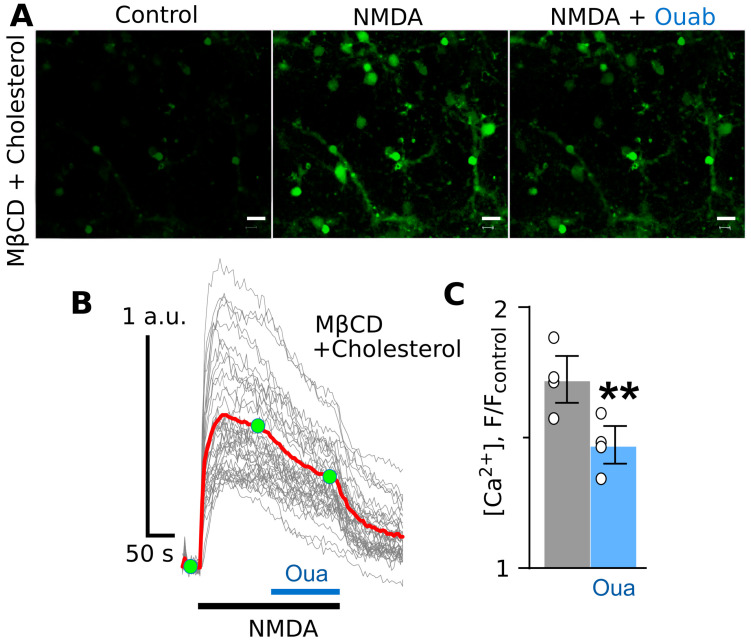
Cholesterol repletion after cholesterol extraction restores the ouabain effect on NMDA-elicited calcium responses in cortical neurons. (**A**) Fluorescent images of neurons in the absence of agonists (control), in the presence of NMDA (100 µM of NMDA and 30 µM of glycine), and in the presence of NMDA and 0.5 nM of ouabain. The bar is 10 µm. (**B**) Fluorescent Ca^2+^ responses of neurons evoked by NMDA (100 µM of NMDA and 30 µM of glycine) obtained via a single experiment and normalized to the fluorescence intensity recorded without NMDA (F/F_control_). Gray lines depict responses of neurons in the optic field, where each line is a response of a single neuron. The red line is the average response of neurons. Moreover, 0.5 nM of ouabain was added on top of the NMDA-evoked response. Green circles indicate the time points of intensity measurements picked for statistics. The bottom bars indicate the protocol of the experiments. (**C**) In the histogram, the average amplitudes of Ca^2+^ responses to NMDA before (grey) and after (blue) ouabain application are compared. Data from each experiment (symbols) and mean values ± S.E.M. are shown. **—the data are significantly different (*p* = 0.006, n = 3, Student’s two-tailed *t*-test).

In the next experiments, we tested whether the restoration of lipid rafts via cholesterol repletion of the plasma membrane could restore the ouabain effect. Indeed, when MβCD-treated neurons were incubated in the basic solution containing 0.5 mM of water-soluble cholesterol for 30 min (Figure 3A), the inhibition of the NMDA-elicited Ca^2+^ response by subnanomolar ouabain was restored (Figure 3B,C).

Obviously, the presence of cholesterol in the plasma membrane is required for ouabain to have effects on NMDA-induced intracellular Ca^2+^ responses in cortical neurons of primary cultures. While neuronal primary cultures represent a reliable and widely utilized experimental object, these preparations usually exhibit a flat neuronal monolayer with random synaptic connections and a minimal presence of glial cells. This approach may be the reason why the results obtained in primary cultures may differ from those in adult neuronal tissue of brain slices [38].

To make sure that the effects of ouabain are not determined based on the peculiarities of the object of investigation, we further performed similar experiments in acute rat brain slices of the L2/L3 layers of the sensorimotor or entorhinal cortex, where excitatory glutamatergic pyramidal neurons represent a majority of the neuronal population [39]. To increase the validity of measurements, astrocytes were stained with SR101 (red fluorescence) to exclude their Ca^2+^ responses from analysis.

**Figure 4:**
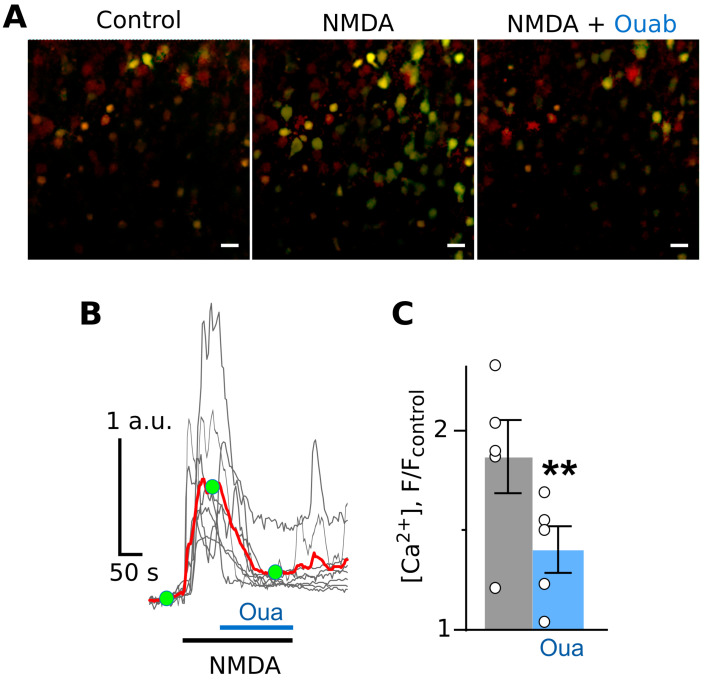
Ouabain causes the attenuation of NMDA-elicited calcium responses in cortical neurons in brain slices. (**A**) Fluorescent images of cells in the absence of agonists (control), in the presence of NMDA (100 µM of NMDA and 30 µM of glycine), and in the presence of NMDA and 1 nM of ouabain. The bar is 10 µm. Green fluorescence corresponds to Ca^2+^-sensitive dye. Red fluorescence corresponds to sulforhodamine staining of astrocytes. The bar is 10 µm. (**B**) Fluorescent Ca^2+^ responses of neurons evoked by NMDA (100 µM of NMDA and 30 µM of glycine) obtained from a single experiment and normalized to the fluorescence intensity recorded without NMDA (F/F_control_). Gray lines depict responses of neurons in the optic field, where each line is a response of a single neuron. The red line is the average response of neurons. Moreover, 1 nM of ouabain was added on top of the NMDA-evoked response. Green circles indicate the time points of intensity measurements picked for statistics. The bottom bars indicate the protocol of the experiments. (**C**) In the histogram, average amplitudes of Ca^2+^ responses to NMDA before (grey) and after (blue) ouabain application are compared. Data from each experiment (symbols) and mean values ± S.E.M. are shown. **—the data are significantly different (*p* = 0.003, n = 5, Student’s two-tailed *t*-test).

On brain slices, an application of 100 µM of NMDA to the bathing solution elicited neuronal Ca^2+^ fluorescence responses, reaching the stable maximum in ~1 min (Figure 4A,B). The addition of 1 nM of ouabain on top of the NMDA-elicited Ca^2+^ response caused it to decline, meaning that the intensities of fluorescence before and after ouabain application became different (Figure 4B,C).

**Figure 5:**
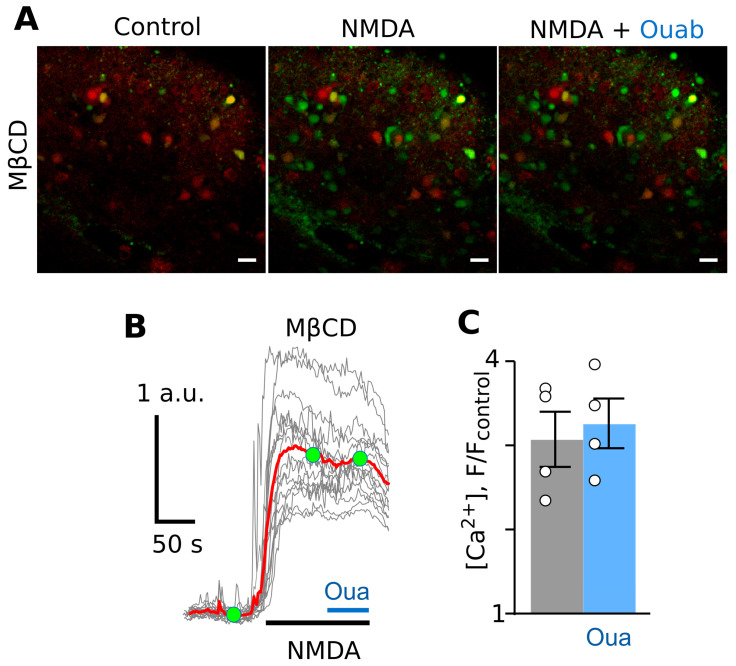
Ouabain does not affect NMDA-elicited calcium response in cortical neurons in brain slices treated with 1.5 mM of MβCD for 5 min. (**A**) Fluorescent images of cells in the absence of agonists (control), in the presence of NMDA (100 µM of NMDA and 30 µM of glycine), and in the presence of NMDA and 1 nM of ouabain. The bar is 10 µm. Green fluorescence corresponds to Ca^2+^-sensitive dye. Red fluorescence corresponds to sulforhodamine staining of astrocytes. The bar is 10 µm. (**B**) Fluorescent Ca^2+^ responses of neurons evoked by NMDA (100 µM of NMDA and 30 µM of glycine) were obtained from single experiment and normalized to the fluorescence intensity recorded without NMDA (F/F_control_). Gray lines depict responses of neurons in the optic field, as each line is a response of a single neuron. The red line is the average response of neurons. Moreover, 1 nM of ouabain was added on top of the NMDA-evoked response. Green circles indicate the time points of intensity measurements picked for statistics. The bottom bars indicate the protocol of the experiments. (**C**) In the histogram, average amplitudes of Ca^2+^ responses to NMDA before (grey) and after (blue) ouabain application are compared. Data from each experiment (symbols) and mean values ± S.E.M. are shown. The data do not significantly differ (*p* = 0.98, n = 4, Student’s two-tailed *t*-test).

After incubation of slices in 1.5 mM of MβCD for 5 min, 100 µM of NMDA caused a neuronal Ca^2+^ response (Figure 5A,B). The application of 1 nM of ouabain, however, did not cause a decrease in the amplitude of the Ca^2+^ response (Figure 5B,C).

Therefore, similar ouabain effects on NMDA-elicited Ca^2+^ responses were observed in cortical neurons in both primary cultures and brain slices. Unfortunately, the direct study of pre-synaptic calcium responses to NMDA using this technique seems to be problematic. However, [Ca^2+^] elevation in pre-synaptic buttons in such experiments is expected because of the presence of autoNMDARs, which may control the residual [Ca^2+^] in the proximity of the vesicle release sites. This outcome may affect the Ca^2+^-dependent spontaneous vesicle release that could be detected as an increase in the frequency of mEPSCs in post-synaptic neurons. We, therefore, verified the most prominent explanation of NMDA and ouabain effects on mEPSC frequency, suggesting that these compounds interact with pre-synaptic NMDARs and NKA involved in the regulation of pre-synaptic intracellular Ca^2+^.

### 3.2. Cholesterol Is Required for Ouabain’s Effect on mEPSC Frequency 

We undertook the study of ouabain effects on the NMDA-induced increase in mEPSC frequency of cortical neurons in both primary cultures and brain slices. For electrophysiological experiments, we used 5 µM of NMDA, a quantity that is lower than the NMDA concentration in imaging experiments, since the activation of NMDARs by 100 µM of NMDA evokes a large integral inward current and current noise, which dramatically decreases the signal-to-noise resolution available for mEPSC detection.

**Figure 6:**
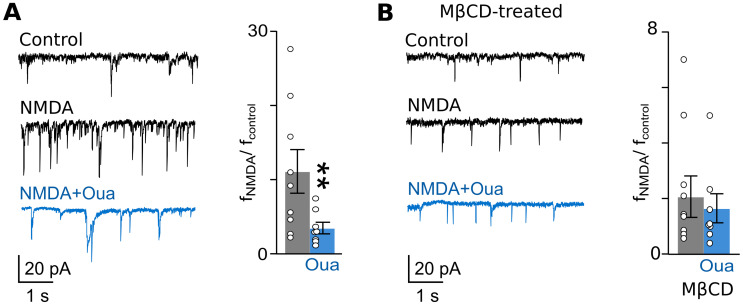
The ouabain effect on mEPSC frequency in cortical neurons of primary culture is abolished via cholesterol extraction from the plasma membrane. (**A**) On the left, traces of mEPSCs recorded at −70 mV in the absence of agonists (control), in the presence of NMDA (5 μM of NMDA and 30 μM of glycine), and in the presence of NMDA and 0.5 nM of ouabain are shown. On the right, relative mEPSC frequencies (f_NMDA_/f_control_) in the presence of NMDA and NMDA and ouabain are compared. Data from each experiment (symbols) and mean values ± S.E.M. are shown. **—the data are significantly different (*p* = 0.0031, n = 8, Student’s two-tailed *t*-test). (**B**) The similar experiment as that shown in panel (**A**) was used, except neurons were treated with 1.5 mM of MβCD for 5 min. The data do not significantly differ (*p* = 0.99, n = 9, Student’s two-tailed *t*-test).

In cortical neurons of primary cultures, an application of 5 µM of NMDA caused an increase in mEPSC frequency in both intact and MβCD-treated neurons (Table 1, Figure 6A). It appeared that in experiments without treatment with MβCD, ouabain decreased the NMDA-induced rise in mEPSC frequency (Figure 6A). In MβCD-treated neurons, ouabain did not affect the NMDA-induced increase in mEPSC frequency (Figure 6B).

**Figure 7:**
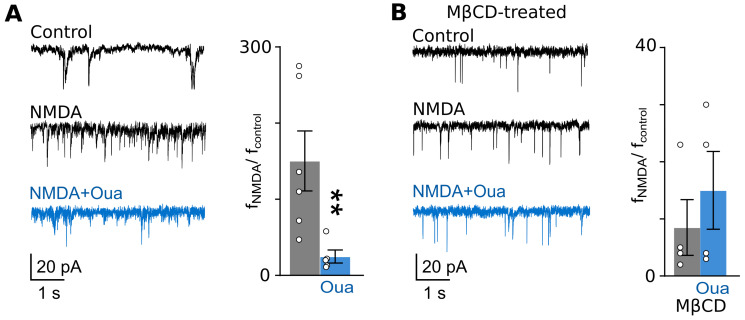
Ouabain’s effect on mEPSC frequency in cortical neurons of brain slices is abolished via cholesterol extraction from the plasma membrane. (**A**) On the left, traces of mEPSCs recorded at −70 mV in the absence of agonists (control), in the presence of NMDA (5 μM of NMDA and 30 μM of glycine), and in the presence of NMDA and 1 nM of ouabain are shown. On the right, relative mEPSC frequencies (f_NMDA_/f_control_) in the presence of NMDA and NMDA and ouabain are compared. Data from each experiment (symbols) and mean values ± S.E.M. are shown. **—the data are significantly different (*p* = 0.0049, n = 6, Student’s two-tailed *t*-test). (**B**) The same experiment as carried out in panel (A), except brain slices were treated with 1.5 mM of MβCD for 5 min. The data do not significantly differ (*p* = 0.98, n = 4, Student’s two-tailed *t*-test).

As for cortical neurons in brain slices, similar experiments revealed, that the addition of 5 µM of NMDA to the bathing solution caused an increase in mEPSC frequency, which reached a stable level after 5–6 min (Table 2, Figure 7A) in both non-treated and MβCD-treated slices. Nevertheless, the ouabain effects under these conditions were found to be different. While without MβCD treatment, 1 nM of ouabain, when NMDA is present, caused a decrease in mEPSC frequency (Figure 7A), ouabain did not change mEPSC frequency in the presence of NMDA in MβCD-treated slices (Figure 7B).

From these experiments, we may conclude that ouabain weakens the NMDA-elicited increase in mEPSC frequency in both experimental objects—primary cultures and brain slices—while it is prevented after cholesterol extraction with MβCD.

It should be further mentioned that MβCD treatment weakened not only the ouabain effect on mEPSC frequency, but also the NMDA-elicited increase in mEPSC frequency, compared to MβCD-untreated neurons in both cultured neurons (Table 1) and brain slices (Table 2). In general, this observation is not surprising because NMDAR desensitization increases in cholesterol-deficient plasma membranes [25,29].

## 4. Discussion

Our experiments demonstrated the inhibitory effect of subnanomolar concentrations of ouabain on NMDA-evoked Ca^2+^ responses of cortical neurons. We hypothesize that NMDAR, NCX, and NKA are the main participants in the process.

An application of the specific NMDAR agonist in our experiments caused intracellular [Ca^2+^] elevation and an increase in mEPSC frequency, which are consistent with the data showing that NMDARs are expressed in both pre- and post-synaptic sites [40]. Their activation leads to an increase in free intracellular [Ca^2+^] and the facilitation of spontaneous vesicle release from pre-synapses, which is manifested in the post-synaptic neuron as the mEPSC frequency increases. We previously demonstrated that loading neurons with the intracellular Ca^2+^ chelator BAPTA completely prevents both the NMDA-elicited increase in mEPSC frequency and ouabain effects [20]. Taken together, these observations support the idea that intracellular Ca^2+^ variations in the neuronal bodies are accompanied by similar events in pre-synaptic terminals.

NCX is the only transport system with enough capacity to counteract large cytosolic Ca^2+^ variations, especially in excitable cells [41], while Ca^2+^-ATPase may rapidly manage small Ca^2+^ variations. Furthermore, the Förster resonance energy transfer study revealed that the major calcium extrusion systems of the plasma membranes of neurons, Ca^2+^-ATPase, and NCX are colocalized in lipid rafts with NMDARs [28]. This colocalization allows rapid interaction between NMDAR and NCX within shared intracellular Ca^2+^ microdomains [25].

Ouabain has a high-affinity binding site on NKA [6,42] but does not interact with NMDAR or NCX. Therefore, ouabain-induced Ca^2+^ handling is most likely determined via NKA signaling that upregulates NCX-mediated Ca^2+^ extrusion rather than inhibition of Ca^2+^ entry. This explanation is consistent with the previous observation that inhibition of NCX prevents the ouabain effects [20]. Therefore, ouabain action most likely depends on colocalization and functional interaction [20] between NMDARs, NCX, and NKA in lipid rafts. This conclusion is supported by the observation that lipid raft disaggregation prevents ouabain effects on intracellular Ca^2+^ and Ca^2+^-dependent spontaneous vesicle release.

**Figure 8:**
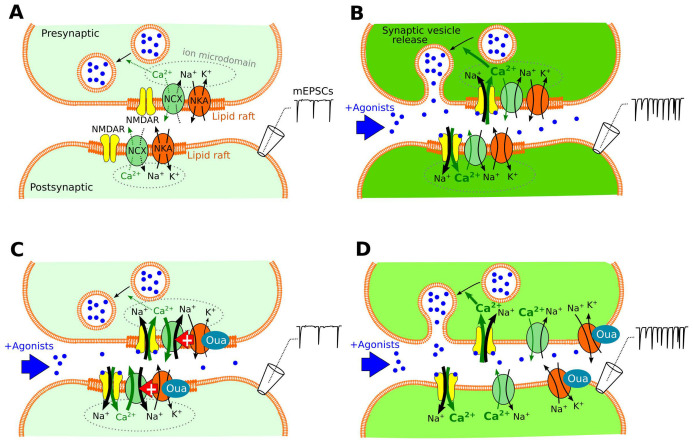
Schematics of data interpretation. (**A**) Control conditions, in which Ca^2+^ entry into neurons and pre-synaptic terminals is compensated via intracellular buffering and extrusion. (**B**) Externally applied agonists of NMDA receptors activate pre- and post-synaptic NMDARs, resulting in intracellular [Ca^2+^] increase, enhanced asynchronous pre-synaptic vesicle release, and a corresponding rise in mEPSC frequency. (**C**) Ouabain (Oua)-bound Na,K-ATPase (NKA) provokes accelerated Ca^2+^-extrusion by the sodium–calcium exchanger (NCX), which handles NMDAR-transferred Ca^2+^ influx to ion microdomains associated with lipid rafts. This process causes rapid lowering of intracellular [Ca^2+^] and related attenuation of synaptic vesicle release, as estimated based on the mEPSC frequency. (**D**) Extraction of cholesterol via MβCD treatment of neurons presumably causes a dissociation between lipid rafts and spatial uncoupling of membrane proteins. This procedure abolishes ouabain-induced potentiation of NCX function by NKA and prevents functional coupling of NMDARs, NCXs, and NKAs within the common ion microdomain. Blue dots denote NMDAR agonists, which are NMDA externally applied, and glutamate in synaptic vesicles. The intensity of the green filling indicates free cytosolic [Ca^2+^].

The step-by-step interpretation of our data is illustrated in Figure 8.

In control conditions (Figure 8A), the Na^+^ transmembrane gradient is maintained by NKA to retain the resting membrane potential, while spontaneous events of Na^+^ and Ca^2+^ entry are fully compensated via calcium extrusion by Ca^2+^-ATPase and NCX. Under these conditions, the intracellular [Ca^2+^] in neurons and pre-synaptic terminals are maintained at ~50 nM [21], which corresponds to a low probability of spontaneous vesicle release exhibited in low mEPSC frequency.

In the case of prolonged presence of externally applied agonist of NMDARs (Figure 8B), Ca^2+^ entry through these ion channels causes an increase in intracellular [Ca^2+^]. NKA is activated via Na^+^ entry, but Ca^2+^ extrusion by NCX cannot handle intracellular Ca^2+^ accumulation. Increased pre-synaptic free Ca^2+^ favors spontaneous vesicle release, which is observed as the increase in the mEPSC frequency.

Subnanomolar ouabain (Figure 8C) somehow enhances Ca^2+^ extrusion by NCX. The exact mechanism of this ouabain effect is not clear and may involve direct NCX–NKA allosteric interaction and ionotropic cooperation within a shared Na^+^ microdomain. Ouabain may also act on NKA in astrocytes; therefore, the functional peculiarities of cultured neurons that lack astrocytes compared to brain slices [38] may determine a difference in ouabain effects. In contrast to brain slices, which have a rather stable tissue cytoarchitecture with astrocyte control of ionic balance in the extracellular microenvironment in tripartite synapses, neurons in primary culture have an obvious deficit of glial cells and lose control of their synaptic microenvironment. 

In spite of the functional diversity between neuronal cultures and brain slices, ouabain eliminated the increase in intracellular Ca^2+^ in neurons evoked by NMDA in both experimental objects. It seems plausible that similar effects of ouabain also occur in the pre-synaptic terminals, since ouabain diminished the increase in mEPSC frequency caused by NMDA. Therefore, the observed ouabain effects probably involve similar mechanisms in both pre- and post-synaptic areas and are not affected by the presence of astrocytes in tripartite synapses in brain slices.

Regardless of the specific mechanism, the functional interaction between NKA and NCX [43], as well as between NCX and NMDAR [25], requires the colocalization of these proteins, which can occur within the lipid rafts. Disaggregation of the lipid rafts via partial cholesterol extraction (Figure 8D) affects the colocalization of raft-associated proteins and, as a consequence, may influence the synaptic transmission through effects on the vesicular release and NKA protein–protein interactions [44]. There is a rough estimation that 1.5 mM of MβCD reduces the concentration of cholesterol in the neuronal membrane by about 20–30% [27], which is enough to disrupt NMDAR association with other proteins [27]. It is also known that this level of cholesterol extraction does not affect NKA ion transport [45] or Ca^2+^ extrusion from cells by NCX and other Ca^2+^-transport mechanisms [46]. Based on these observations, we may suggest that the main effects of MβCD treatment in our experiments are the solubilization of lipid rafts [28] and the loss of colocalization between NMDAR, NCX, and NKA. This procedure probably prevents local functional interaction between NKA and NCX; therefore, ouabain-bound NKA in the absence of lipid rafts does not influence Ca^2+^ extrusion. Under these conditions, ouabain also does not decrease mEPSC frequency, as shown by the lack of its effect on pre-synaptic free [Ca^2+^].

Cholesterol repletion after depletion restored ouabain effects, suggesting that re-association with lipid rafts restores the functional interaction between Ca^2+^ entry via NMDARs, Ca^2+^ extrusion by NCX, and NKA. This result further supports our conclusion regarding the mechanism of ouabain action in glutamatergic synapses: these proteins are located in both pre- and post-synapses; therefore, ouabain demonstrated effects on both cytosolic bulk Ca^2+^ and spontaneous synaptic vesicle release.

It should be mentioned that MβCD treatment decreased NMDA-elicited Ca^2+^ responses in cultures but did not do so brain slices. This mismatch probably reflects worse conditions for cholesterol extraction in brain tissue than in cell cultures. Regardless of this contradiction, MβCD abolished the effect of ouabain on calcium responses in both experimental objects. Therefore, the ability of MβCD to prevent the ouabain effects cannot be attributed to MβCD-induced weakening of NMDA-elicited Ca^2+^ responses.

Taken together, our observations favor the following conclusions: (i) Ouabain-induced inhibition of the NMDA-elicited Ca^2+^ response involves both the pre- and post-synapses. Moreover, (ii) the presence of astrocytes in the tripartite synapse is not required for ouabain effects. Finally, (iii) ouabain action requires the integrity of lipid rafts, which are responsible for the colocalization and functional interaction of NMDAR-elicited Ca^2+^ entry, NCX-mediated Ca^2+^ extrusion, and NKA modulation of NCX. While these conclusions seem plausible considering the physiological focus of our experiments, they do not allow us to shed light on the molecular bases of NMDAR, NCX, and NKA interplay. This issue could be addressed in further investigations using biochemical approaches.

Thus, we demonstrated that NMDAR, NCX, and NKA are involved in the regulation of intracellular residual [Ca^2+^] in both pre- and post-synaptic neurons, and this process depends on the integrity of lipid rafts. The residual [Ca^2+^] of pre-synaptic buttons determines the probability of transmitter release. Therefore, any small changes in the calcium entry or its handling could cause large consequences for synaptic strength [47] because the pre-synaptic [Ca^2+^] regulates both synchronous and asynchronous synaptic vesicle release [48]. In agreement with previous reports [20,21,22,23], cardiotonic steroid ouabain causes a decrease in evoked Ca^2+^ responses in our experiments. This outcome is the reason why low nanomolar ouabain weakens the long-term potentiation of synaptic transmission [23]. Both Ca^2+^ entry by NMDARs [49] and Ca^2+^ extrusion by NCX [50] are indispensable players of neuronal Ca^2+^ homeostasis and synaptic plasticity. The pharmacological effects of some widely used medicines, like tricyclic antidepressants, on NMDARs robustly depend on both the modulation of NMDAR Ca^2+^-dependent desensitization by NCX [51] and the integrity of lipid rafts [32]. Intracellular [Ca^2+^] variations, therefore, have a pleiotropic effect on many physiological mechanisms, including NMDAR desensitization, synaptic plasticity-related changes, and other numerous Ca^2+^-dependent processes. Hence, the modulation of intracellular residual [Ca^2+^] in lipid rafts by cardiotonic steroids and other modulators may interfere with the pharmacological actions of neurological medicines.

## Figures and Tables

**Table 1:** Absolute values of mEPSC frequencies in primary cultures of cortical neurons (events per second).

	Control	NMDA	NMDA and Oua
No treatment	3.1 ± 0.48 s^−1^ (n = 10)	19.5 ± 4.0 s^−1^ (n = 10)	9.9 ± 2.0 s^−1^ (n = 8)
MβCD	1.9 ± 0.4 s^−1^ (n = 8)	2.6 ± 0.6 s^−1^ (n = 8) **	2.1 ± 0.5 s^−1^ (n = 8)

**—the data are different (*p* = 0.001) to those of MβCD-untreated neurons. Data were compared using Student’s two-tailed *t*-test.

**Table 2:** Absolute values of mEPSC frequencies in cortical neurons in brain slices (events per second).

	Control	NMDA	NMDA and Oua
No treatment	0.25 ± 0.07 s^−1^ (n = 12)	16.8 ± 2.4 s^−1^ (n = 6)	8.1 ± 1.0 s^−1^ (n = 5)
MβCD	1.3 ± 1.0 s^−1^ (n = 4)	4.9 ± 2.6 s^−1^ (n = 4) **	7.8 ± 3.4 s^−1^ (n = 4)

**—the data are different (*p* = 0.003) to those of MβCD-untreated neurons. Data were compared using Student’s two-tailed *t*-test.

## Data Availability

The original contributions presented in the study are included in the article; further inquiries can be directed to the corresponding author.
